# Male-female comparison of vasomotor effects of circulating hormones in human intracranial arteries

**DOI:** 10.1186/s10194-024-01933-w

**Published:** 2024-12-11

**Authors:** Jacob C. A. Edvinsson, Irena Grubor, Aida Maddahi, Lars Edvinsson

**Affiliations:** 1 Department of Clinical Sciences, Faculty of Medicine, Lund University, Getingevagen 4, Lund, 22185 Sweden; 2 Department of Neurosurgery, University Hospital, Lund, Sweden

**Keywords:** Estrogen, Oxytocin, Vasopressin, Progesterone, Testosterone, Adrenomedullin, Amylin, CGRP

## Abstract

**Background:**

The purpose of this study was to examine whether there are sex differences in vasomotor responses and receptor localization of hormones and neuropeptides with relevance to migraine (vasopressin, oxytocin, estrogen, progesterone, testosterone, amylin, adrenomedullin and calcitonin gene-related peptide (CGRP)) in human intracranial arteries.

**Methods:**

Human cortical cerebral and middle meningeal arteries were used in this study. The tissues were removed in conjunction with neurosurgery and donated with consent. Vasomotor responses of arteries, after exposure to hormones or neuropeptides, were recorded using a wire myograph. Immunohistochemistry was performed to examine the expression and localization of their receptors within human intracranial arteries.

**Results:**

Vasopressin showed the strongest contractile responses, followed by oxytocin and progesterone. CGRP displayed the strongest vasodilatory response when compared to adrenomedullin, amylin, testosterone and estrogen. No significant differences were observed in vasomotor responses between male and female arteries. The vasomotor effects were supported by the presence of corresponding receptors in the vascular smooth muscle cells. Estrogen receptors (ERα and ERβ), progesterone receptor (PR), vasopressin 1a receptor (V1aR), and the oxytocin receptor (OTR) were expressed in the walls of both cerebral arteries overlying the cerebral cortex and intracranial arteries of the dura mater. ERα, V1aR, and PR were found to be localized in both smooth muscle cells and endothelium, whereas OTR was exclusively located within the smooth muscle cells.

**Conclusions:**

Hypothalamic, sex hormones and the pancreas hormone (amylin) receptors are expressed in the human intracranial artery walls. The vasomotor responses revealed no sex differences, however contractile responses to vasopressin was higher and more potent in MMA compared to CCA when pooling data from both sexes. Overall, the hormones estrogen, progesterone and oxytocin, which drop in circulating levels at onset of menstruation, only showed modest vasomotor responses as compared to CGRP. This suggests that their role in inducing menstrual migraine attacks is not directly related to vasomotor responses.

**Graphical Abstract:**

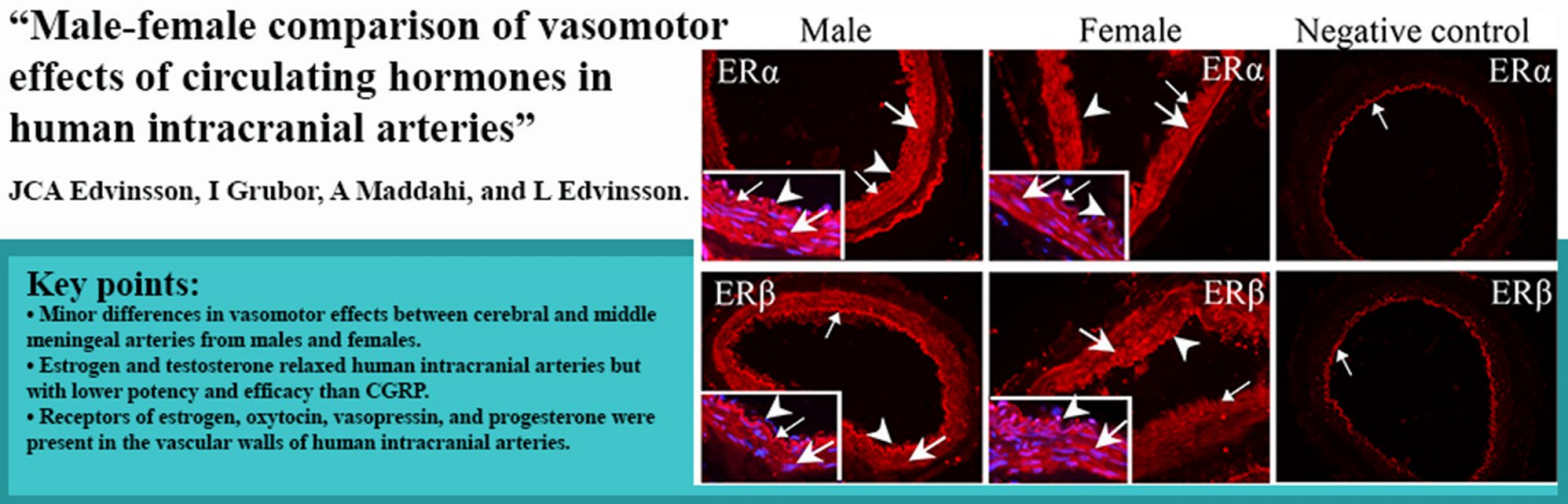

**Supplementary Information:**

The online version contains supplementary material available at 10.1186/s10194-024-01933-w.

## Introduction

Male-female differences are evident in cerebrovascular physiology and pathophysiology [1]. These sex differences impact diagnosis, disease progression and treatment of cerebrovascular disorders [2]. Yet few studies have investigated possible male-female differences in cerebrovascular regulation and fewer still have examined sex differences in intracranial arteries of humans [3].

We have previously shown sex differences in the vascular reactivity of human brain arteries [3]. Vasoconstrictor responses to angiotensin II and endothelin were lower in cerebral arteries from women as compared to those from men. There is a predominance of migraine in females, and the attacks often coincide with the initiation of menstruation and its dynamic alterations in circulating hormone levels [4]. The pathophysiology of migraine is linked to the trigeminovascular system (TGVS), with particular interest on the potential effects of vasoactive peptides and reproductive hormones on human middle meningeal artery (MMA), an intracranial artery emanating from the externa carotid artery, and cerebral arteries originating in the internal carotid artery [5].

It is frequently advocated that dilatation of meningeal arteries is a key factor in induction of migraine attacks [6, 7]. Therefore, we asked the question if sex hormones and hypothalamus hormones show expression of specific receptors in the walls of intracranial arteries that can mediate vasomotor responses. Further, after the rise of calcitonin gene-related peptide (CGRP) as a successful therapeutic target in migraine treatment, the less studied CGRP-family members adrenomedullin and amylin are of interest as potential new targets [8].

As the hypothalamus has been postulated to be of great importance for the generation of migraine [9], the hypothalamic hormones vasopressin and oxytocin are of interest when studying the vascular aspects of migraine. It is well-known that the global female population suffers disproportionally from migraine when compared to men, therefore it has been suggested that sex hormones play an important part in menstrual-related migraine [10]. In this context it is important to note that menstrual-related migraine attacks are associated with reductions in the circulating levels of estrogen, progesterone and oxytocin [5, 11].

The aim of the present study was to examine the direct vasomotor responses of oxytocin, vasopressin, testosterone, estrogen, and progesterone in fresh isolated human intracranial arteries and to compare whether are differences between males and females. In addition, the expression of relevant hormone receptors in the vessel walls were examined using immunohistochemistry. Human artery samples from male and female patients undergoing neurosurgical procedures were donated for this study. The donated arteries were identified as either middle meningeal arteries (MMA) or cortical cerebral arteries (CCA). The latter is protected by the BBB while the former is not [12].

## Methods

All experiments were carried out in accordance with national laws and guidelines and approved by the ethical committee at Lund University (LU-818-01). The arteries were collected, and the experiments performed, from January 2021 to May 2024.

### Human arteries

Fresh human samples of MMA (5 males, aged 35–75 years and 4 females, aged 40–86 years) and cortical cerebral artery (CCA) branches (5 males, aged 44–82 years and 5 females, aged 54–71 years) were acquired, with oral and written consent, from patients undergoing neurosurgery at the University Hospital of Lund. The tissue samples were placed in cold Dulbecco’s modified Eagle’s medium (DMEM, Gibco, Invitrogen, Carlsbad, CA, USA) and immediately transported to the laboratory.

The samples were placed in a cold, oxygenated Krebs buffer solution composed of NaCl 119 mM, NaHCO_3_ 15 mM, KCl 4.6 mM, MgCl_2_ 1.2 mM, NaH_2_PO_4_ 1.2 mM, CaCl_2_ 1.5 mM and glucose 5.5 mM; pH 7.4. The arteries were carefully dissected from the brain and surrounding connective tissue.

### Myography

The arteries were cut into 1–2 mm long ring segments that were placed in parallel tissue baths of cold Krebs buffer solution aerated with oxygen enriched with 5% CO_2_, with a resulting pH of 7.4 [13].

Each segment of MMA (internal diameter 0.2–1.25 mm) and cerebral artery (internal diameter 0.25–0.65 mm) was mounted on a pair of stainless-steel wires (40 μm caliber) in an arterial myograph (Mulvaney–Halpern, Denmark). One wire was connected to a micrometer screw, allowing for fine adjustment of the vascular tone by varying the distance between the wires. The other wire was connected to a force displacement transducer, paired with an analogue-digital converter (ADInstruments, Oxford, UK). Data was recorded on a computer using a PowerLab unit and LabChart (ADInstruments, Oxford, UK).

In contractile experiments, the artery segments were submerged in oxygenated Krebs solution at + 37 °C. In relaxation experiments, a Krebs solution enriched with 30 mM K^+^ (+ 37 °C) was used to attain a stable level of pre-contraction.

The tension on each segment was normalized to 90% of the internal circumference that a fully relaxed vessel would have under a transmural pressure of 100 mmHg. A reference value for the maximal contractile capacity (100%) of each segment was determined by replacing an equimolar part of the NaCl in the buffer solution with KCl (60 mM K^+^). Segments with a reference value lower than 0.5 mN were excluded from the experiments.

The integrity of the endothelium was confirmed by pre-contracting the segment with a buffer containing 30 mM K^+^ and adding substance P [10^− 9^–10^− 7^ M] to evoke a transient, concentration-dependent dilatation of > 20%. Arteries not responding were excluded from inclusion in the study. In addition, CGRP, a key molecule in migraine with strong vasodilator effect, was used to compare the relaxant responses of the hormones.

Cumulative concentration-response curves were obtained for agonists in individual artery segments tested in parallel (using up to 8 tissue baths, which allows for individual tests). Each segment was only exposed to one of the sex hormones in separate segments to avoid interactions. By running 8 separate tissue baths in parallel we avoided any possible interaction between the hormones or CGRP.

### Immunohistochemistry

Dissected CCA and MMA artery segments (4 males, 38–60 years of age, and 4 females, 54–62 years of age) were fixed in 4% paraformaldehyde in phosphate-buffered saline (PBS, Sigma Aldrich, pH 7.2) overnight at + 4 °C. To ensure cryoprotection, the fixated arteries were submerged in increasing concentrations of sucrose (from 10 to 25%) in Sorensen’s phosphate buffer overnight. The following day, the segments were embedded in Tissue-Tek ^®^ O.C.T. ™ Compound Tissue TEK (Sakura Finetek Europe B.V., The Netherlands). The frozen blocks were cryosectioned at 10 μm using a cryostat (Microm Cryo Star HM 560) and collected on microscope slides (Superfrost^™^, Merck Chemicals and Life Science, Sweden). The slides were stored at -20 ºC until use.

Sections were washed and permeabilized in PBS containing 0.25% Triton-X (PBS-T) for 15 min. Then, the sections were incubated with primary antibodies (for details, see Table 1) in humidity chambers at + 4 ºC overnight. The following day, the sections were washed in PBS-T, 2 × 15 min, to remove excess primary antibodies. Subsequently, the sections were incubated with appropriate secondary antibodies (Table 1) for one hour in a dark room (RT). Excess secondary antibodies were washed off using PBS-T, 2 × 15 min. Lastly, a cover glass was mounted on the sections using Vectashield mounting medium containing 4’,6-Diamidino-2-phenylindole (DAPI; Vector Laboratories, Burlingame, CA, USA).

**Table 1a:** Primary antibodies used for immunohistochemistry

Name and product code	Dilution	Host	Immunogen	Supplier	References
Estrogen Receptor α (sc-7207)	1:100	Rabbit	Amino acids 2-185 of ER α of human origin	Santa Cruz Biotechnology, CA, USA	Warfvinge et al. *J Headache Pain 2020*
Estrogen Receptor β (ab288)	1:100	Mouse	C-terminal of human RAMP1	Abcam, Cambridge, UK	Warfvinge et al. *J Headache Pain 2020*
Progesterone Receptor (MA5-12658)	1:50	Mouse	PgR from human endometrial carcinoma grown in athymic mice	Thermo Scientific, IL, USA	Maddahi et al.*J headache and pain 2023*.
Oxytocin Receptor (ab87312)	1:400	Goat	Synthetic peptide to the C terminal	Abcam, Cambridge, UK	Warfvinge et al. *J Headache Pain 2020*.
Vasopressin receptor 1 A(ab18775)	1:250	Rabbit	Synthetic peptide to rat AVPR1A aa 155–173	Abcam, Cambridge, UK	Albee et al. *Open Biol 2018.* Maddahi et al.*J headache and pain 2022.*

**Table 1b:** Secondary antibodies used for immunohistochemistry

Conjugate	Dilution	Against	Supplier
Alexa 488	1:100	Anti-goat	Thermo Scientific, IL, USA
FITC	1:100	Anti-mouse	Jackson Immunoresearch, West Grove, PA, USA
Alexa 594	1: 200	Anti-goat	Thermo Scientific, IL, USA
FITC	1:100	Anti-rabbit	Jackson Immunoresearch, West Grove, PA, USA

Experiments were performed in triplicate for each human to ensure reproducibility. Negative controls for each secondary antibody were included by omitting the primary antibody to evaluate non-specific binding and autofluorescence. The sections were examined in a light and epifluorescence microscope (Nikon 80i, Tokyo, Japan) equipped with a Nikon DS-2MV camera. Figure montages were processed using Adobe Photoshop CS3 (v8.0, Adobe Systems, Mountain View, CA, USA). The selectivity and specificity of the antibodies have been verified before and references are listed in Table 1.

### Calculations

For substances where a complete concentration-contractile response curve could be obtained, the maximum response (E_max_ %) in each artery segment was measured as the percentage of the initial contractile response to 60 mM K^+^. Where appropriate, the maximum dilatory response (E_max_ %) was expressed as % dilation relative to the level of precontraction with 30 mM K^+^. The pEC_50_ values were calculated using log (agonist) vs. response variable slope in the GraphPad Prism software (Version 9.3.1, San Diego, California, USA). For substances where it was not feasible to test higher concentrations to obtain a plateau response, the response observed with the highest concentration used (i.e. 10 µM) was considered as the E_max_ [14].

Statistical analyses were performed with non-parametric t-test when comparing two groups, and two-way ANOVA when comparing three groups. All data are presented as mean ± standard error of the mean (S.E.M). Statistical significance was accepted at *P* < 0.05. In results, n represents the number of patients.

### Chemicals

Estrogen, testosterone, oxytocin, and CGRP were obtained from Sigma Aldrich, Merck Life Science AB, Sweden. Vasopressin, progesterone, and substance P were obtained from Bio-Techne Ltd, United Kingdom.

## Results

### Myography

#### Contractile responses

**Fig. 1:**
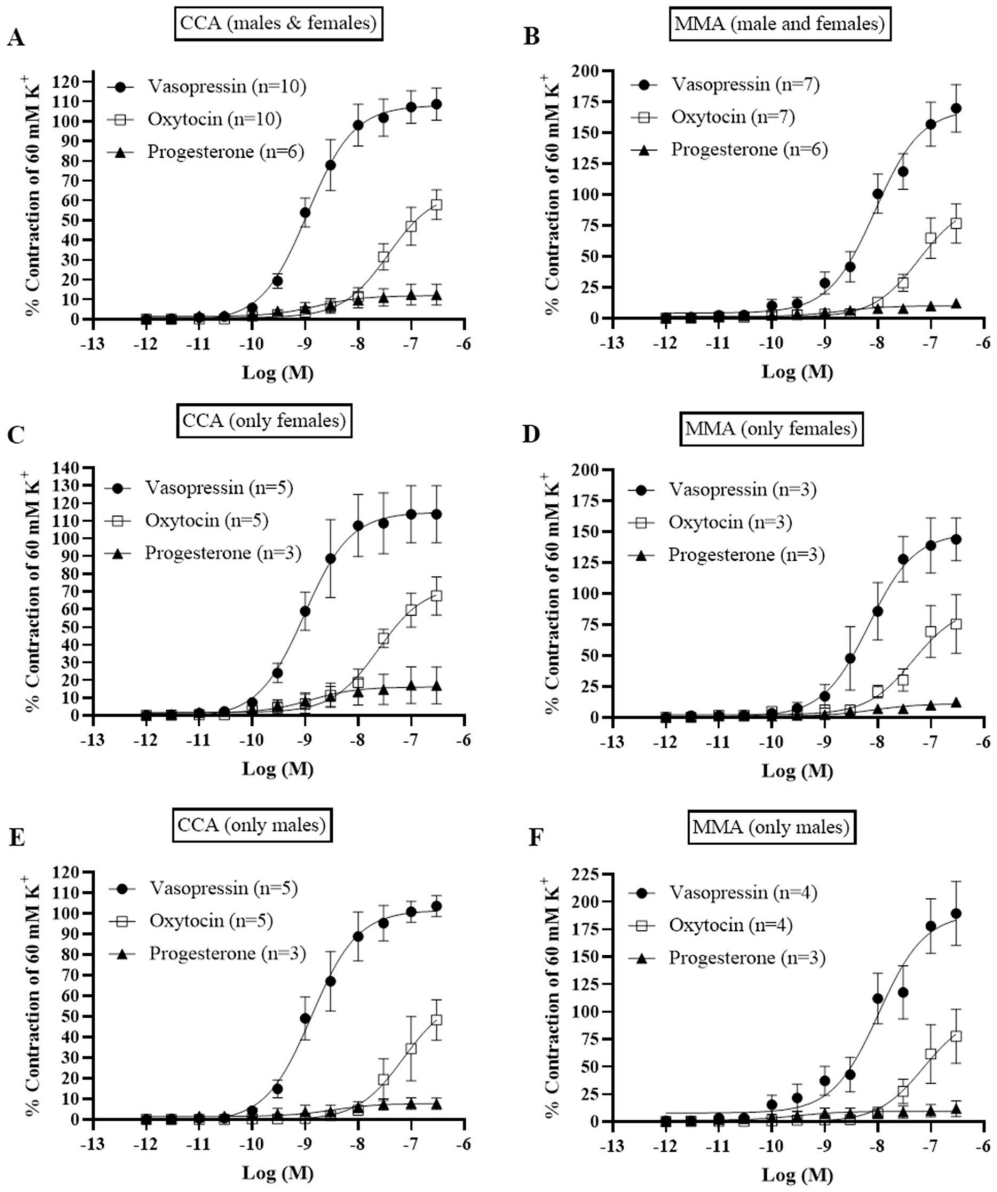
Contractile responses in fresh segments of intracranial arteries, from males and females, after addition of vasopressin, oxytocin, and progesterone. The data from cerebral arteries in **a**) are obtained from *n* = 6 patients (5 males and one female). The data from MMA in **b**) are obtained from two males and three females. Data points represent mean ± SEM. Significance for the effect of hormones compared to control was determined with unpaired parametric t-test. *P* < 0.05 was considered significant

All vessel segments responded with strong contraction upon exposure to 60 mM potassium buffer. After wash-out and normalized tension, concentration-dependent contractile responses were recorded in CCA and MMA after administration of increasing concentrations of vasopressin, oxytocin, and progesterone (Fig. 1).

The strongest contractions recorded in CCA (Fig. 1A), and MMA (Fig. 1B) were noted with vasopressin, displaying an E_max_ of 108.1 ± 8.2% and 169.3 ± 19.2%, respectively. The maximum contraction was significantly stronger in MMA versus the CCA (*p* < 0.05), likewise vasopressin displayed a higher potency in MMA compared to CCA (Table 2). Oxytocin had an observed E_max_ of 57.8 ± 7.6% in the CCA and 76.6 ± 15.9% in the MMA. As shown in Fig. 1, there was no sex difference between CCA and MMA either in efficacy or in potency. Progesterone had the lowest observed contractile effects with an E_max_ of 12.3 ± 5.3% in the CCA and 12.0 ± 3.3% in the MMA. The calculated pEC_50_ showed no difference (Table 2). Previously, the contractile responses to vasopressin and oxytocin have been shown to be mediated via a vasopressin receptor [15]. The pooled (males & females) data for CCA (Fig. 1A) suggests a higher potency when compared to the pooled MMA (Fig. 1B) data for vasopressin. This difference does not seem to be related to sex as we observed no significant discrepancy when comparing male versus female data. No significant difference was observed between the vascular types (CCA and MMA) or sex in the oxytocin and progesterone results (Fig. 1).

#### Relaxant responses

Pre-contraction with 30 mM K^+^ buffer resulted in a stable contractile tone of all the artery segments, during which concentration-dependent dilatations were recorded after the cumulative addition of CGRP, estrogen, or testosterone.

**Fig. 2:**
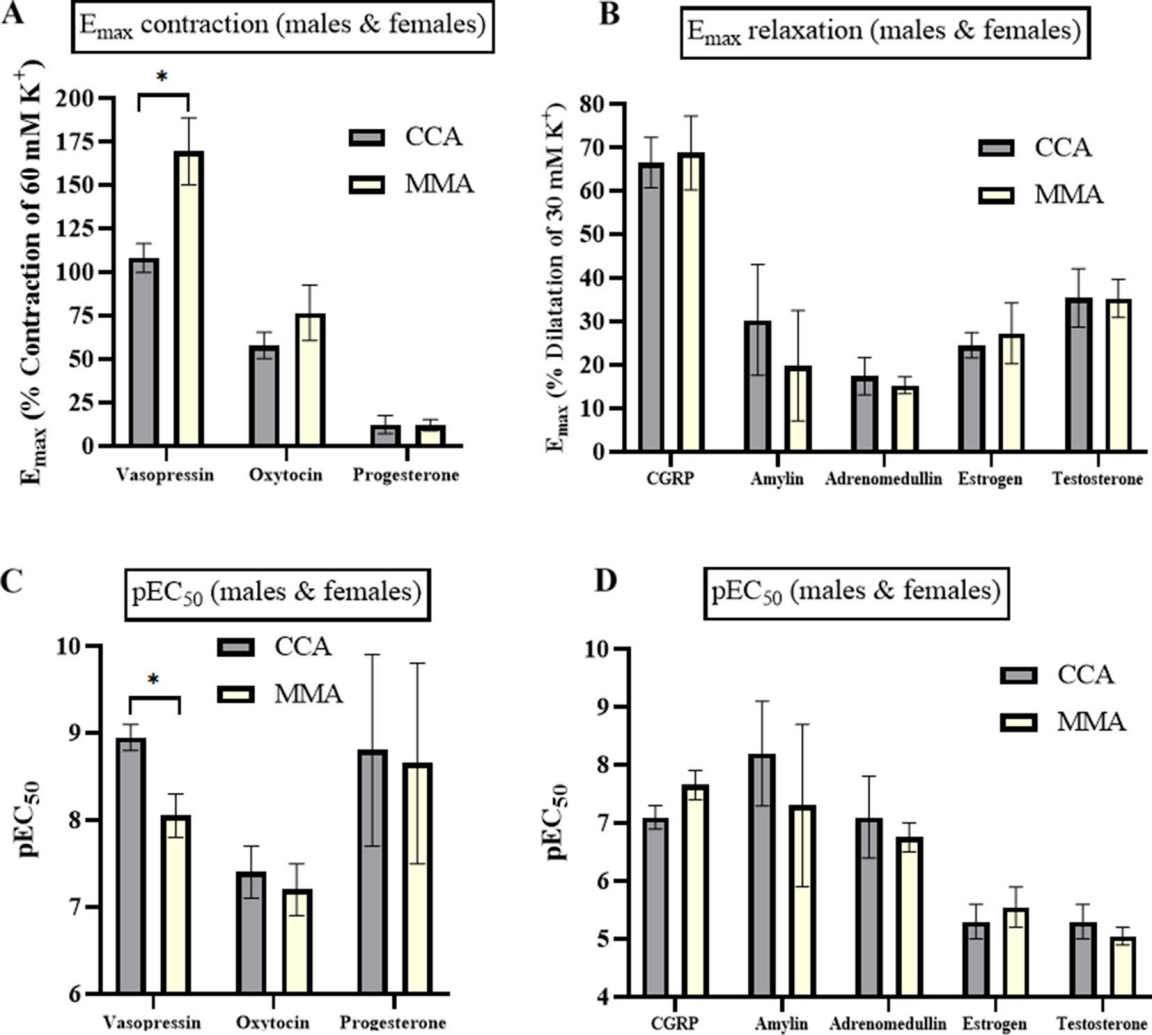
Emax and pEC_50_ values, data presented as mean ± SEM. The maximal dilatory effects of CGRP, amylin, adrenomedullin, 17β-estradiol, and testosterone were presented as % dilatation of the precontraction with 30 mM K^+^ (E_max_). pEC_50_ abbreviates the logarithm of the drug concentration producing half of the maximum contraction or dilatation. Data points represent mean ± SEM, *n* = 3–10 for cerebral arteries, and *n* = 3–9 for MMA. Significance was determined with unpaired parametric t-test. *P* < 0.05 was considered significant

The most efficacious and potent dilatations were observed with CGRP, displaying an E_max_ of 66.5 ± 5.8% in CCA and 68.7 ± 8.5% in MMA (Fig. 2). There were no significant differences observed for CGRP in either potency or efficacy between male and female arteries (Table 2). In contrast, amylin and adrenomedullin showed weak responses with lower pEC_50_ values compared to their effects on their respective receptors (Fig. 2) [16].

**Fig. 3:**
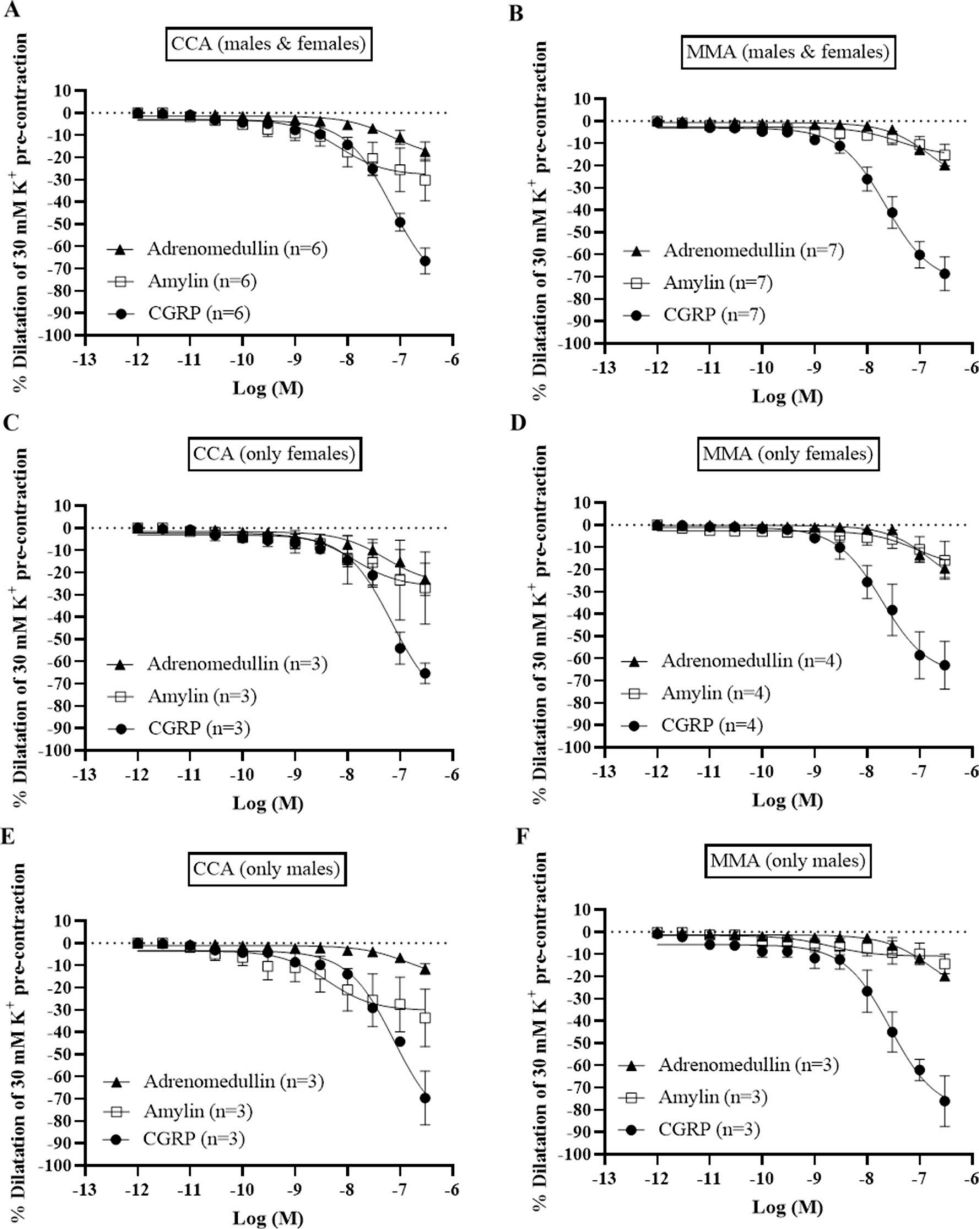
Vasodilatory effects of CGRP, Adrenomedullin and Amylin were investigated in segments of intracranial arteries from males and females by adding cumulative concentrations from 10^− 12^ M to 10^− 5^ M to vessels pre-contracted with 30 mM K^+^. A summary of the data collected in cortical cerebral arteries (CCA) and middle meningeal arteries (MMA) is presented (**A**-**B**). A subdivision for female and male subjects was also made to observe any sex specific differences (**C**-**F**). Data points represent mean ± SEM. Significance was determined with unpaired parametric t-test. *P* < 0.05 was considered significant

Estrogen displayed relaxant responses having an E_max_ of 24.5 ± 2.9% in CCA, and 27.3 ± 7.0% in MMA (Fig. 3), with no difference in potency (Table 2). The relaxant response to estrogen has been shown to be mediated via the ERα receptor [17]. Testosterone had a slightly stronger relaxant effect than estrogen with an E_max_ of 35.4 ± 6.7% in the CCA, and 35.3 ± 4.4% in the MMA. However, there was no difference in maximum relaxant responses between vessel types neither in E_max_ nor in pEC_50_ (Fig. 3). The difference in efficacy between estrogen and testosterone was not significant, nor was there any difference between male and female arteries.

### Immunohistochemistry

**Fig. 4:**
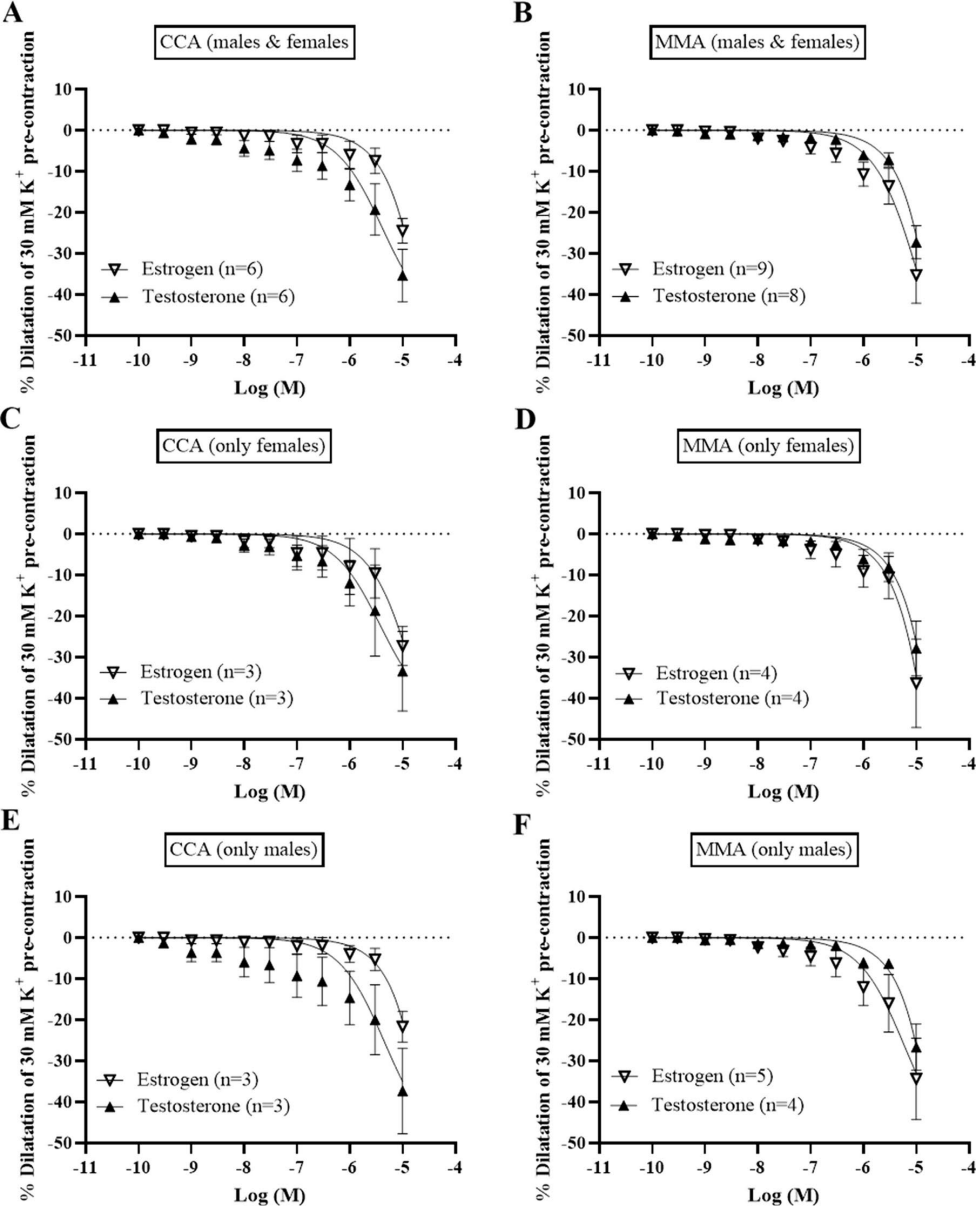
Vasodilatory effects of estrogen and testosterone on human isolated arteries. A summary of the data collected in cortical cerebral arteries (CCA) and middle meningeal arteries (MMA) is presented (**A**-**B**). A subdivision for female and male subjects was also made to observe any sex specific differences (**C**-**F**). Significance was determined with unpaired parametric t-test. *P* < 0.05 was considered significant. Data points represent mean ± SEM, and n = the number of subjects

**Fig. 5:**
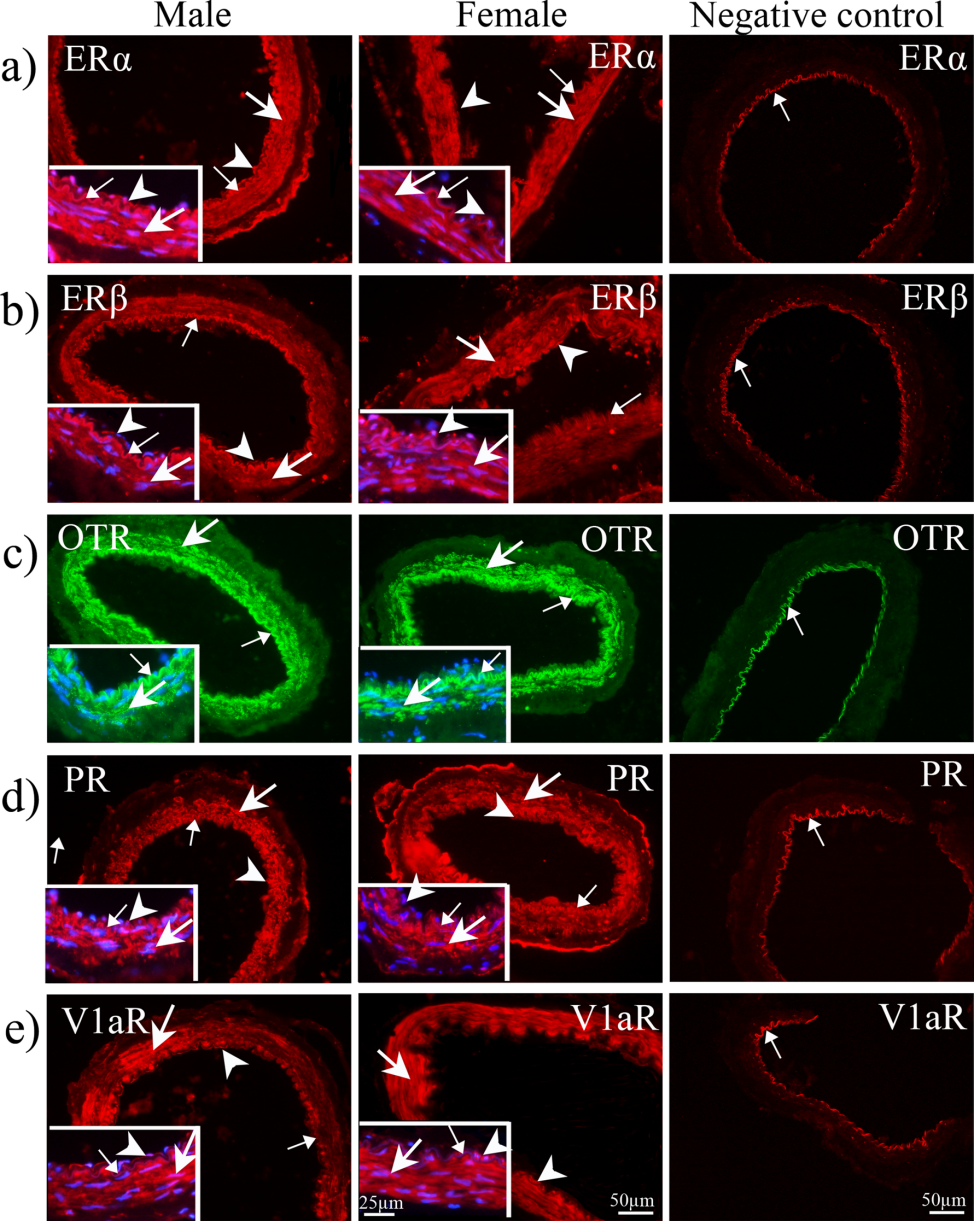
Immunohistochemistry of ERα (**a**), ERβ (**b**), OTR (**c**), PR (**d**), and V1aR (**e**) in human cortex artery from males and females. Immunoreactivity of ERα and ERβ observed in both SMCs (thick arrows) and endothelial (arrowheads) cells, whereas OTR was only observed in SMCs (thick arrows). Immunoreactivity of PR and V1aR was also observed in SMCs (thick arrows) and endothelium (arrowheads). Blue color represents nuclear staining with DAPI in higher magnification of images. The internal elastic lamina was observed as autofluorescence in male, female, and negative control (thin arrows)

The localization of ERα, ERβ, OTR, PR and V1aR in human arteries were examined with immunohistochemistry, using well characterized antibodies (Table 1). The immunoreactivity for ERα, ERβ, PR and V1aR were mainly localized in the cytoplasm of the vascular smooth muscle cells (SMCs), and partly in endothelial cells, in CCA of both sexes (Fig. 4). OTR immunoreactivity was observed in the cytoplasm of SMCs (Fig. 4C), while we found no immunoreactivity for OTR in endothelial cells. We did not observe any difference in expression, or localization, of the hormone receptors in CCA and MMA (Fig. 5). Similarly, there was no obvious discrepancy in arterial expression between males and females for the investigated receptors. Negative controls displayed no ERα, ERβ, OTR, PR and V1aR immunoreactivity in the walls of arteries, only the internal elastic lamina displayed auto-fluorescence (Fig. 4).

## Discussion

This study examined the vascular responses to hormones and neuropeptides in male and female intracranial arteries. With immunohistochemistry we found expressions of ERα, ERβ, OTR, PR and V1aR, which were located primarily in the VSMCs. Previously, the canonical CGRP receptor has been demonstrated and characterized in the VSMCs of intracranial arteries, with both immunohistochemistry and myograph studies [18].

Vasopressin displayed strong vasoconstriction and was the only hormone showing a significant difference between artery types, where vasopressin in MMA had higher efficacy and potency compared to CCA (Fig. 2). However, no significant difference was observed for vasopressin between sexes. The expression of the V1a receptor was found in VSMCs of both MMA and CCA. Previous work on human middle cerebral arteries also showed strong and potent vasoconstriction that was blocked by a specific V1 receptor antagonist [19, 20].

Another important hypothalamic hormone is the closely related oxytocin (differs by 2 amino acids relative to vasopressin) and its receptor, which have widespread and important roles both in the brain and in the periphery [17, 21, 22]. In the present study, we demonstrated the presence of the OTR in VSMCs of CCM and MMA of both males and females. Oxytocin is a vasoconstrictor of human intracranial arteries, and blockade experiments revealed its functional presence in a pharmacology study which agrees with previous experiments [23]. The closely related hormone vasopressin also induced strong constriction via its receptor, V1aR [24], which is expressed in VSMCs and the endothelium of human intracranial arteries (Figs. 4 and 5). The expression of V1aR was more prominent in the VSMCs when compared with endothelium, which agrees with its strong vasoconstrictor effect of human intracranial arteries.

The sex hormone estrogen is a weak vasodilator and the observed reduction in circulating levels prior to the onset of menstruation would have minor vascular implication on the tone of the MMA in vivo [25]. However, estrogen has been postulated to be involved in the regulation of 5-hydroxytryptamine, and in turn CGRP, which may have secondary vascular consequences [26].

At the start of menstruation, the hormones estrogen, oxytocin and progesterone all show marked reductions in circulation levels to their lowest monthly levels [5]. The collective effects of these 3 hormones with relaxant and contractile effects would result in minor vasomotor effects. The circulating levels of these 3 hormones are low relative to their respective pEC_50_, and in menstrual migraine they are at their lowest circulating levels. This suggests that the induction of menstrual migraine attacks is not related to a direct vasomotor response to the reduced levels of these hormones [5].

Pharmacologically, estrogen primarily acts via ERα to cause vasorelaxation in human intracranial arteries [27]. Interestingly, we did not observe a significant difference in functional responses between human male and female intracranial arteries. One plausible explanation may be that the vessels came from women of near post-menopausal age. Whereas age-related reductions in plasma levels of hormones are well characterized, much less is known about the effects of ageing on the vascular receptors to sex hormones [28, 29]. It was observed that the expression of endothelial ERα was reduced by a third in peripheral veins of postmenopausal women, but whether the receptor expression was down regulated due to lower plasma levels of estrogen or related to age could not be established [30]. Like the estrogen receptors, progesterone receptors are also widespread in vascular beds [31], and in the present study we observed PR in both endothelium and VSMCs of intracranial arteries from both females and males.

The vasomotor effects of estrogen, oxytocin, and vasopressin were in the present study recorded at concentrations ten times higher than their normal circulating plasma levels which would refute a vasomotor effect to induce the menstrual related migraine attacks in particular since their levels are at their lowest circulating levels precisely at the time of the menstruation onset [32–34].

Our results do not demonstrate a significant difference neither in maximum nor in potency to CGRP between arteries from male or female patients. Recently, de Vries et al. observed that the CGRP induced relaxation was more potent/stronger in female MMA at the age 15–49 versus > 50 years of age [35]. As demonstrated previously the CGRP receptor are mainly found in the VSMC while CGRP is expressed in C-fibers innervating the adventitial layer [18, 36]. Similarly, we found that the responses of hypothalamus and sex hormones did not differ significantly between male and female CCM and MMA. Due to the paucity of obtained tissue specimens, it was not possible to run more quantitative analysis of the different receptors. However, the protein examination of the vessels using immunohistochemistry revealed their major localization to the VSMCs. In addition, the functional results are in concert with the protein data. The responses to other members of the CGRP family (amylin, adrenomedullin and calcitonin) have been reported to have pEC_50_ values at their respective receptors of about 9.0–10.0 [37]. We observed only weak relaxations of amylin and adrenomedullin when compared to CGRP, suggesting that they are putatively active at the canonical CGRP receptor. Recent data on the human MMA agree and are supported by qPCR analysis of the mRNA of CLR, CTR and RAMPs [35]. In addition, previous analysis of calcitonin has demonstrated only minor vasodilation at high concentrations [16].

The discrepancy between male and female prevalence of migraine is likely explained by fluctuations of sex hormones during the menstrual cycle [5]. At menopause, hormonal levels are more irregular for many years, and the hormone receptors very slowly change into the male phenotype. Therefore, it may take long time for the females to reach a male phenotype.

As the female samples used in this study was predominantly from patients assumed to be menopausal, this could explain why no significant difference between the sexes could be observed. However, these results indicate that there may be a difference in the pain response mediated by activation of the trigeminovascular reflex between the sexes. In support of this, previous studies have suggested that females have more reported migraine-triggers and a lower pain-threshold when compared to males [5, 38].

In conclusion, while the hormones are not produced in intracranial arteries, their receptors are clearly expressed in the VSMCs of CCA and MMA, with no difference between males and females. This study has shown that the hypothalamus and sex hormones have effects on vascular tonus in human intracranial arteries, albeit with minor impact at physiological concentrations. Since the menstrual-related migraine attacks are associated with reductions in estrogen, oxytocin and progesterone, the data suggests that hormone induced vasodilatation is not likely to be the initiating trigger. In agreement, experimental studies have shown that induced CGRP release is not modified by concomitant exposure to either of these hormones [17, 23, 24]. The reason behind hormone-related migraine attacks needs further analysis.

### Highlights

- Estrogen and testosterone relaxed human intracranial arteries but with lower potency and efficacy than CGRP.
- Oxytocin, vasopressin, and progesterone had contractile effects on human intracranial arteries.
- There were minor differences in vasomotor effects between cerebral and middle meningeal arteries from males and females.
- Receptors of estrogen, oxytocin, vasopressin, and progesterone were present in the vascular walls of human intracranial arteries.

### Limitations

**Fig. 6:**
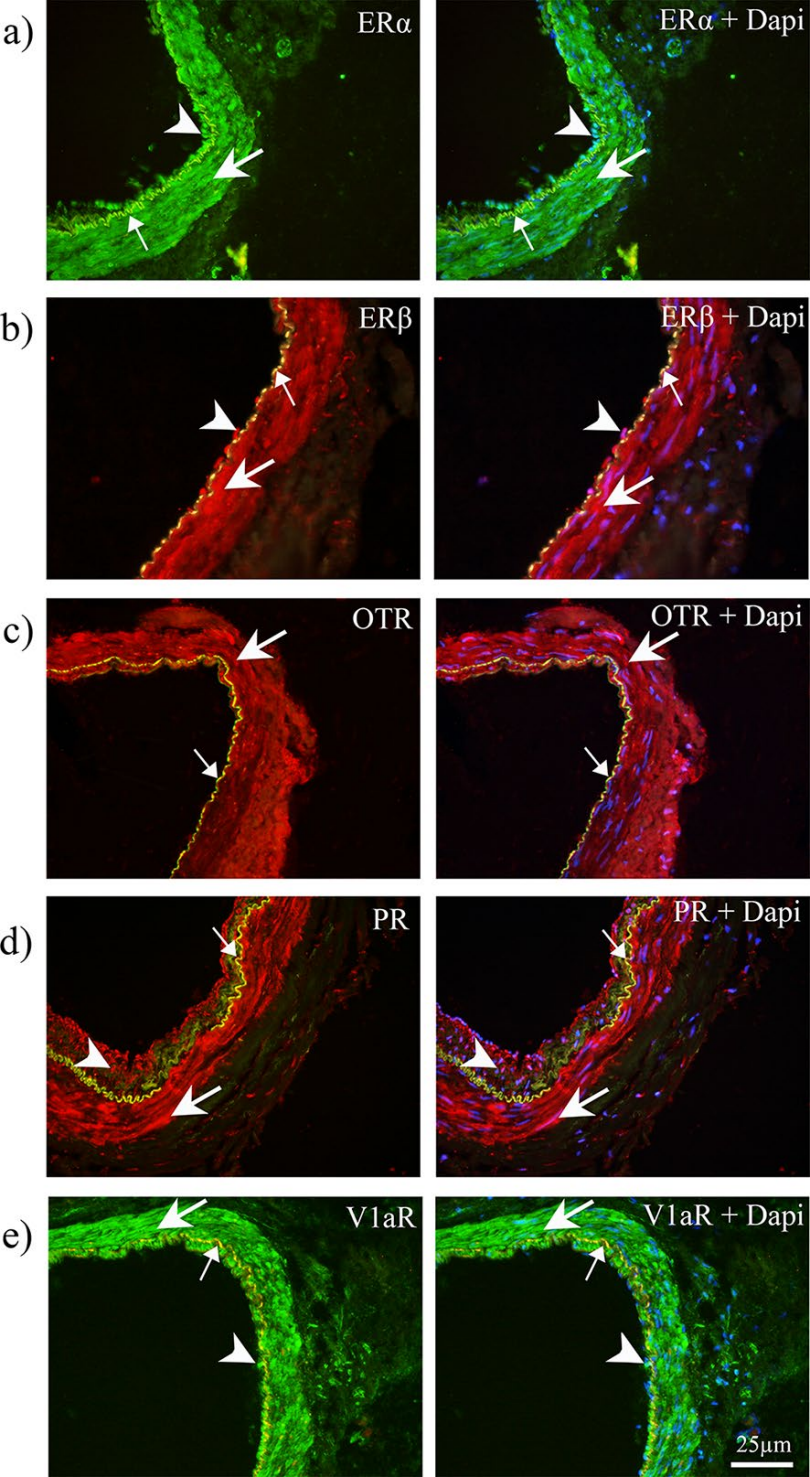
Immunohistochemistry of ERα (**a**), ERβ (**b**), OTR (**c**), PR (**d**), and V1aR (**e**) in human dura artery from females. ERα, ERβ, PR, and V1aR immunoreactivities were localized in both SMCs (thick arrows) and EN (arrowheads) whereas OTR immunoreactivity was observed in the SMCs (arrows). The internal elastic lamina was observed as auto fluorescent in yellow color (thin arrows)

We used fresh human arteries removed from patients undergoing neurosurgery. They were immediately placed in cold DMSO and transported to the laboratory within 60 min, thus handled as quickly as possible. However, the subjects were under medication and anesthesia, which may impact vasomotor responses. The patient ages were mainly considered older (see Supplementary Table 3), which influences hormonal levels and possibly their response ex vivo. Further, we did not have access to individual patient journals and could therefore not investigate possible interfering factors, such as menopausal status or headache history (See Fig. 6).

## Electronic supplementary material

Below is the link to the electronic supplementary material.

Supplementary Material 1

## Data Availability

No datasets were generated or analysed during the current study.

## Abbreviations

BBB: Blood-brain barrier
CGRP: Calcitonin gene-related peptide
CNS: Central nervous system
DAPI: 4’,6-Diamidino-2-phenylindole
DMEM: Dulbecco’s modified Eagle’s medium
EN: Endothelium
ER: Estrogen receptor
MMA: Middle meningeal artery
OTR: Oxytocin receptor
PBS: Phosphate-buffered saline
PBS-T: Phosphate-buffered saline with 0.25% Triton-X
PNS: Peripheral nervous system
PR: Progesterone receptor
SMC: Smooth muscle cell
V1aR: Vasopressin receptor 1a
