# Supplementary material for: Male-female comparison of vasomotor effects of circulating hormones in human intracranial arteries
Source: J Headache Pain. 2024 Dec 11;25(1):216. doi: 10.1186/s10194-024-01933-w (PMC11633024; doi:10.1186/s10194-024-01933-w)
Supplement: Supplementary file 1 — Supplementary Material 1 [file 10194_2024_1933_MOESM1_ESM.docx]

|  | **Males & Females** | | | | | |
| --- | --- | --- | --- | --- | --- | --- |
|  | **CCA** | | | **MMA** | | |
|  | n | E_max_ ± SEM (%) | pEC50, 95% CI | n | E_max_ ± SEM (%) | pEC50, 95% CI |
| CGRP | 6 | 66.5 ± 5.8 | 7.3 – 6.9 | 7 | 68.7 ± 8.5 | 7.9 - 7,4 |
| Amylin | 6 | 30.3 ± 12.7 | 9.1 – 7.3 | 7 | 19.8 ± 12.7 | 8.7 – 5.9 |
| Adrenomedullin | 6 | 17.4 ± 4.3 | 7.8 – 6.4 | 7 | 15.3 ± 1.9 | 7.0 – 6.5 |
| Estrogen | 6 | 24.5 ± 2.9 | 5.6 – 5.0 | 9 | 27.3 ± 7.03 | 5.9 – 5.2 |
| Testosterone | 6 | 35.4 ± 6.7 | 5.6 – 5.0 | 8 | 35.3 ± 4.4 | 5.2 – 4.9 |
| Vasopressin | 10 | 108.1 ± 8.2 | 9.1 – 8.8 | 7 | 169.3 ± 19.2 | 8.3 – 7.8 |
| Oxytocin | 10 | 57.8 ± 7.6 | 7.7 – 7.1 | 7 | 76.6 ± 15.9 | 7.5 – 6.9 |
| Progesterone | 6 | 12.3 ± 5.3 | 9.9 – 7.7 | 6 | 12.0 ± 3.3 | 9.8 – 7.5 |

**Supplementary Table 1.** E_max_ values and pEC_50_ values of each experimental drug separated into groups corresponding to artery type. Data is presented as E_max_ ± SEM (%), and pEC_50_ values are molar within a 95% confidence interval (CI). n = the number of individual patients.

|  | **CCA** | | **MMA** | |
| --- | --- | --- | --- | --- |
|  | **Males (%)** | **Females (%)** | **Males (%)** | **Females (%)** |
| CGRP | 69.7 ± 12.1 [3] | 65.4 ± 4.6 [3] | 69.7 ± 11.4 [3] | 63.1 ± 10.7 [4] |
| Amylin | 33.7 ± 12.9 [3] | 27.0 ± 16.2 [3] | 19.9 ± 4.5 [3] | 16.0 ± 8.5 [4] |
| Adrenomedullin | 11.7 ± 2.4 [3] | 23.2 ± 7.4 [3] | 14.5 ± 1.5 [3] | 19.6 ± 4.0 [4] |
| Estrogen | 21.7 ± 3.8 [3] | 27.3 ± 4.8 [3] | 34.4 ± 9.9 [5] | 36.4 ± 10.7 [4] |
| Testosterone | 37.3 ± 10.4 [3] | 33.4 ± 9.7 [3] | 26.7 ± 5.6 [4] | 27.9 ± 6.7 [4] |
| Vasopressin | 103.6 ± 5.1 [5] | 113.7 ± 16.2 [5] | 189.2 ± 29.1 [4] | 143.7 ± 17.4 [4] |
| Oxytocin | 48.2 ± 9.8 [5] | 67.4 ± 10.8 [5] | 77.5 ± 24.7 [4] | 75.3 ± 23.6 [4] |
| Progesterone | 7.7 ± 2.8 [5] | 17.0 ± 10.4 [5] | 11.7 ± 7.2 [4] | 12.3 ± 1.8 [4] |

**Supplementary Table 2.** Maximum responses of each experimental drug separated into groups corresponding to sex and artery type. Data is presented as E_max_ ± SEM [n], where n = the number of individual patients. There were no significant differences between males and females for either agonist neither in CCA nor in MMA.

| **CCA** | | **MMA** | |  |
| --- | --- | --- | --- | --- |
|  |  |  |  |  |
| **Birth year** | | **Birth year** | |  |
| Female | Male | Female | Male |  |
| 1953 | 1949 | 1938 | 1960 |  |
| 1960 | 1946 | 1945 | 1949 |  |
| 1970 | 1980 | 1984 | 1967 |  |
| 1961 | 1942 | 1982 | 1946 |  |
| 1950 | 1963 |  | 1955 |  |

**Supplementary Table 3.** Sex and birth year for the donated human arteries used in this study.
